# Molecular Farming of Pembrolizumab and Nivolumab

**DOI:** 10.3390/ijms241210045

**Published:** 2023-06-12

**Authors:** Michael C. Stark, Anna M. Joubert, Michelle H. Visagie

**Affiliations:** Department of Physiology, School of Medicine, Faculty of Health Sciences, University of Pretoria, Private Bag X323, Pretoria 0031, South Africa; mr.mcstark@gmail.com (M.C.S.); annie.joubert@up.ac.za (A.M.J.)

**Keywords:** cancer, immune checkpoint inhibitors, pembrolizumab, nivolumab, molecular farming

## Abstract

Immune checkpoint inhibitors (ICIs) are a class of immunotherapy agents capable of alleviating the immunosuppressive effects exerted by tumorigenic cells. The programmed cell death protein 1 (PD-1)/programmed death-ligand 1 (PD-L1) immune checkpoint is one of the most ubiquitous checkpoints utilized by tumorigenic cells for immune evasion by inducing apoptosis and inhibiting the proliferation and cytokine production of T lymphocytes. Currently, the most frequently used ICIs targeting the PD-1/PD-L1 checkpoint include monoclonal antibodies (mAbs) pembrolizumab and nivolumab that bind to PD-1 on T lymphocytes and inhibit interaction with PD-L1 on tumorigenic cells. However, pembrolizumab and nivolumab are costly, and thus their accessibility is limited in low- and middle-income countries (LMICs). Therefore, it is essential to develop novel biomanufacturing platforms capable of reducing the cost of these two therapies. Molecular farming is one such platform utilizing plants for mAb production, and it has been demonstrated to be a rapid, low-cost, and scalable platform that can be potentially implemented in LMICs to diminish the exorbitant prices, ultimately leading to a significant reduction in cancer-related mortalities within these countries.

## 1. Introduction

Cancer is one of the leading causes of mortality worldwide and accounted for almost 10 million deaths in 2021 [1]. According to the World Health Organization, lung, colorectum, liver, stomach and breast cancer resulted in the majority of these cancer-related mortalities [2]. Approximately 70% of these deaths occurred in low- and middle-income countries (LMICs), reflecting a significant gap in the availability of comprehensive therapeutics [1,3]. Previous literature has shown that the accessibility of comprehensive cancer treatments is less than 15% in LMICs, but greater than 90% in high-income countries [2]. With the incidence of cancer diagnoses estimated to increase by 47% by 2040, there is an increasing requirement to improve the efficacy of current treatments while promoting accessibility for cancer patients in LMICs [1].

The conventional armamentarium of cancer treatment includes the use of surgery, radiation, and particularly chemotherapy [4]. However, chemotherapeutic agents are not specific for tumorigenic cells, and thus also damage non-tumorigenic cells in the body, resulting in a plethora of side effects, including fatigue, diarrhea, myelosuppression, neutropenia and in some cases, death [5]. Thus, a considerable amount of research has focused on the development of novel targeted therapies, including monoclonal antibodies (mAbs) that bind specific tumor surface proteins and stimulate the immune system against tumorigenic cells [6,7]. Immunotherapy is a promising cancer therapy capable of activating the immune system or attenuating the immunosuppressive effects of tumorigenic cells [8,9]. Immune checkpoint inhibitors (ICIs) are one class of immunotherapeutics that have demonstrated high clinical success rates and function by inhibiting immune checkpoints between tumorigenic cells and cluster of differentiation 8 (CD8) T lymphocytes [10]. Furthermore, the most frequently targeted immune checkpoint for cancer therapy includes the programmed cell death protein 1 (PD-1) and the programmed death-ligand 1 (PD-L1) pathway [11]. PD-1 is a receptor found on T lymphocytes and binds to its ligand PD-L1 commonly overexpressed in several types of cancer including lung-, pancreatic-, gastric- and ovarian cancer [11,12]. Ultimately, the binding of PD-1 to PD-L1 prevents the proliferation of T lymphocytes, inhibits cytokine production and results in the induction of apoptosis via the inhibition of survival- and growth signaling pathways including the Phosphoinositide 3-kinase (PI3K)/protein kinase B (Akt)- and the rat sarcoma (RAS) pathway [13,14]. Therefore, PD-1 is an integral component involved in the progression and survival of tumorigenic cells [14].

The most frequently used ICIs currently on the market include pembrolizumab (Keytruda^®^) and nivolumab (OPDIVO^®^), which are both anti-PD-1 mAbs [15]. These two mAbs bind with high affinity to PD-1 on T lymphocytes and thereby inhibit the binding of PD-L1 on tumorigenic cells [11,12]. The latter leads to the activation of T lymphocytes, resulting in the induction of apoptosis in tumorigenic cells through T-cell-mediated cytotoxicity [11]. Nonetheless, the extortionate price of pembrolizumab and nivolumab attributable to the mammalian cell production platform used to produce these two therapies makes them hardly accessible in LMICs [16,17]. The mammalian cell production platform involves the use of recombinant deoxyribonucleic acid (DNA) technology wherein transgenic mice and cells are produced expressing the mAb gene [15,18].

Approximately 95% of synthesized antibodies used for the treatment of rheumatoid arthritis, Crohn’s disease and several types of cancer are produced in mammalian cells despite the high production costs, biosafety concerns and high investment capital needed [19]. Thus, the development of cost-effective and scalable production platforms that can be easily implemented in LMICs is a necessity. The utilization of molecular farming for the development of mAbs is gaining immense interest in the biopharmaceutical field, since this production method is significantly safer and more economical when compared to other platforms [8]. *Nicotiana benthamiana* is a common plant species used for the transient and stable expression of mAbs and is potentially the most rapid and cost-efficient platform capable of mitigating the socio-economic burden brought upon by these two therapies [16,17]. This review highlights the recent advances in molecular farming for the production of pembrolizumab and nivolumab and elucidates the role of the PD-1/PD-L1 axis in cancer. The findings in this paper provide novel insights regarding the use of plants as bioreactors and the potential for a low-cost and high-throughput recombinant mAb production platform.

## 2. PD-1/PD-L1 Axis in Cancer

The ability of tumorigenic cells to evade detection and subsequent destruction by the immune system is a hallmark of cancer [20]. One method employed by tumorigenic cells is upregulating the expression of PD-L1, a protein involved in immune checkpoint control [11]. PD-L1 is a 33-kDa type I transmembrane protein which consists of 290 amino acids with an immunoglobulin (Ig) and IgC extracellular domain [14]. Furthermore, PD-L1 plays an integral role in non-tumorigenic processes as well, particularly by suppressing the immune system in pregnancy, autoimmune diseases and hepatitis by binding to its co-inhibitory receptor, found on T lymphocytes [20]. The increased expression of PD-L1 is exploited by tumorigenic cells, and the literature indicates that there is a significant correlation with advanced disease and malignancy in skin, bladder, breast, liver, pancreatic, ovarian and lung cancer [21]. Recently, a study investigating the effect of PD-L1 expression on the survival rate of 877 non-small cell lung cancer (NSCLC) patients demonstrated that PD-L1 overexpression directly correlates with poor survival due to increased proliferation and survival of tumorigenic lung cells by preventing T-cell-mediated immune responses [22]. Thus, due to the inhibition of T-cell-mediated immune responses, considerable research has taken place to identify the extrinsic and intrinsic factors regulating PD-L1 expression in tumorigenic cells [23].

The main extrinsic factors regulating PD-L1 expression are the pro-inflammatory cytokines, interferon gamma (IFN-γ) and tumor necrosis factor alpha (TNF-α) [13,24,25]. IFN-γ is produced and secreted by T cells, macrophages and natural killer (NK) cells in the tumor microenvironment and substantially increases PD-L1 expression in tumorigenic cells by activating protein kinase D isoform 2 (PKD2) [14,25,26]. Upon activation, PKD2 interacts with proteins involved in pro-survival signaling pathways, particularly extracellular signal-regulated kinase (ERK), which stimulates the nuclear factor kappa B (NF-κB) transcription factor, leading to the upregulation of PD-L1 gene expression [27,28]. TNF-α is another cytokine known to increase PD-L1 expression by activating the constitutive photomorphogenesis 9 (COP9) signalosome 5 (CSN5) and NF-κB cell signaling pathway [29]. In addition, CSN5 is also capable of inhibiting the ubiquitination of PD-L1 and consequently increases PD-L1 expression [29]. Overall, TNF-α and IFN-γ play crucial roles in the inducible expression of PD-L1; however, a myriad of other intrinsic factors, including the genomic amplification of the PD-L1 gene and the abnormal expression of certain transcription factors (hypoxia-inducible factor 1-alpha (HIF1-α), myelocytomatosis proto-oncogene (MYC) and NF-κB) which stimulate PD-L1 expression are known to have significantly greater effects [20].

**Figure 1:**
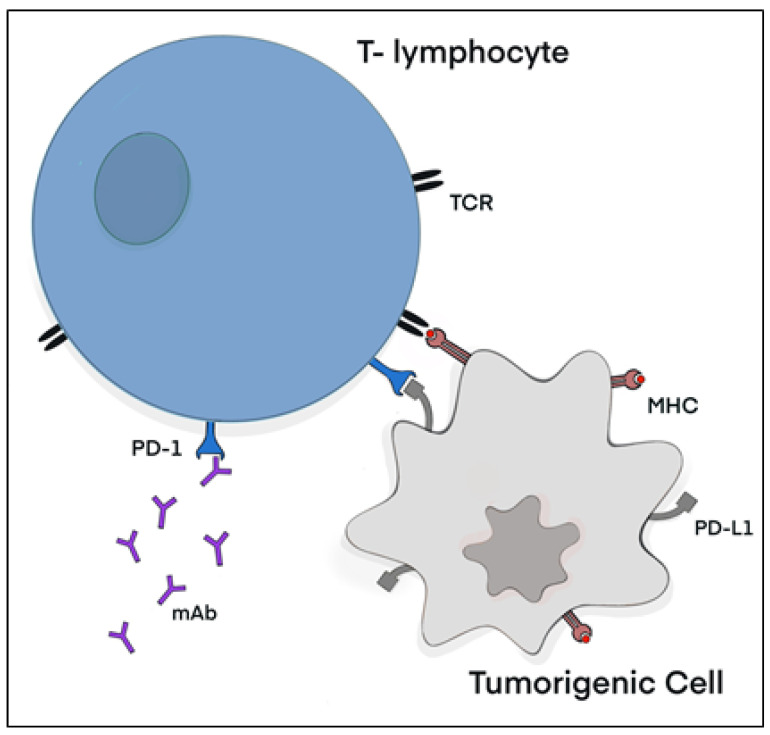
Schematic representation of the PD-1/PD-L1 checkpoint and anti-PD-1 mAbs. The identification of tumorigenic cells by T lymphocytes depends on the recognition of tumor-associated antigens displayed on the major histocompatibility complex (MHC) class I protein through the binding of the T-cell receptor (TCR) [11]. Upon TCR activation, the T lymphocyte will initiate T-cell mediated cytotoxicity, ultimately leading to the induction of apoptosis in the tumorigenic cell [11]. However, tumorigenic cells upregulate the expression of PD-L1, which binds to its receptor PD-1 on T lymphocytes, consequently inhibiting TCR signaling and thus T-cell mediated cytotoxicity [35]. Anti-PD-1 mAbs inhibit the interaction of PD-1 to PD-L1, allowing TCR activation and signaling [36]. Image designed by MC Stark using Microsoft^®^ Office PowerPoint (Microsoft Office enterprise 2007, 2006 Microsoft Corporation, Redmond, WA, USA).

Intrinsically, the upregulation of PD-L1 in tumorigenic cells is attributed to the genomic amplification of chromosome 9p24.1, which houses the PD-L1 gene [23]. This amplification has been noted in several types of cancer, with the highest frequency of PD-L1 copy number alterations (CNAs) evident in primary mediastinal B-cell lymphoma, Hodgkin lymphoma and triple-negative breast cancer (TNBC) at approximately 63%, 40% and 29%, respectively [30,31,32]. In addition to this, several transcription factors are known to upregulate PD-L1 expression, particularly MYC, NF-κB and the signal transducer and activator of transcription 3 (STAT3) [21,23,33]. Furthermore, MYC is abnormally expressed in 70% of all cancers and increases PD-L1 expression by directly stimulating transcription by binding to the promoter region [21,23,33]. The inhibition of MYC in tumorigenic cells results in a significant reduction of PD-L1 expression at both the transcriptomic and proteomic level [33]. Previous literature has shown that RAS drives PD-L1 expression through the RAS/Mitogen-activated protein kinase/ERK kinase (MEK)/ERK cell signaling pathway [23,34]. Thus, these intrinsic factors contribute to the increased expression of PD-L1, and therefore play a major role in the survival and progression of cancer [21,23]. On the whole, PD-L1 overexpression is utilized by tumorigenic cells to exert an immunosuppressive effect by binding to its receptor (PD-1) displayed on T lymphocytes (Figure 1).

PD-1 is a 55-kDa type I transmembrane protein possessing 288 amino acids with an extracellular IgV domain and is displayed on the surface of CD8^+^ cytotoxic T lymphocytes (CTLs) [14,37]. CTLs are one of the most important effectors in the anti-tumor immune response, mediated by the binding of the TCR to an antigen displayed on the MHC [11,38]. Ultimately, this interaction results in the induction of apoptosis in tumorigenic cells mediated by the secretion of perforin and granzyme B from CTLs [39]. However, the binding of PD-1 to PD-L1 inhibits the cytotoxic response and, as a consequence, tumorigenic cells are capable of evading immune surveillance and destruction [13].

**Figure 2:**
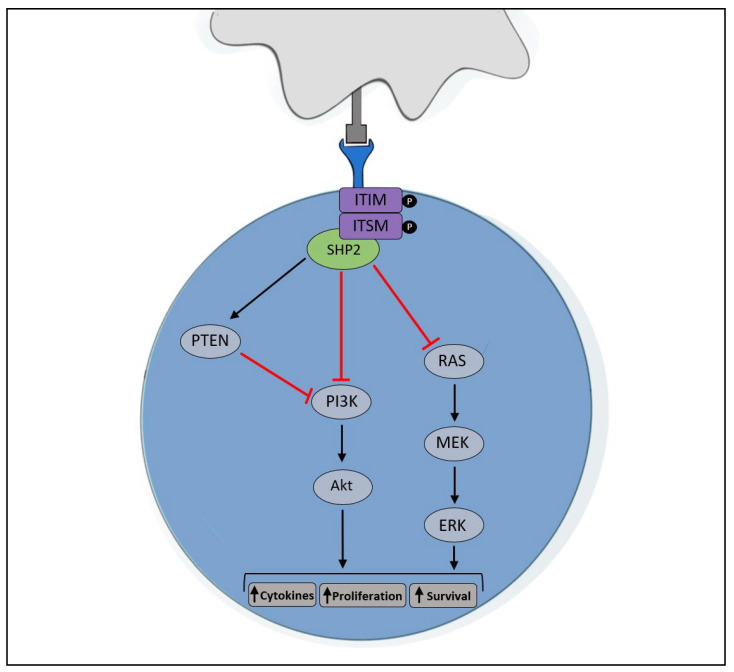
PD-1 cell signaling pathway. The binding of PD-L1 to PD-1 results in the phosphorylation of ITIM and ITSM, subsequently leading to the recruitment of SHP2, which goes on to inhibit the PI3K/Akt and RAS/MEK/ERK signaling pathways. Altogether, this leads to the inhibition of proliferation and cytokine production and the induction of apoptosis [35,40]. In addition, SHP2 stimulates phosphatase and tensin homolog (PTEN), which dephosphorylates PI3K—inhibiting its activity [45]. Image designed by MC Stark using Microsoft^®^ Office PowerPoint (Microsoft Office enterprise 2007, 2006 Microsoft Corporation, Redmond, WA, USA).

The interaction between PD-1 and PD-L1 induces a myriad of downstream effects in CTLs, consequently resulting in apoptosis and the inhibition of proliferation and cytokine production [35]. The binding of PD-1 to PD-L1 induces the phosphorylation of immunoreceptor tyrosine-based inhibitory motif (ITIM) and immunoreceptor tyrosine-based switch motif (ITSM) in the PD-1 intracellular domain, consequently leading to the recruitment of Src homology phosphatase 2 (SHP2) which dephosphorylates several crucial proteins (PI3K and RAS) in the TCR signaling pathway (Figure 2) [35,40]. Consequentially, the activation events of NF-κB and B-cell lymphoma-extra-large (Bcl-xL) are repressed, and thus the production of several cytokines, including interleukins (ILs), TNF-α and IFN-γ are inhibited [41,42]. Moreover, the decrease in the anti-apoptotic activity of Bcl-xL ultimately leads to apoptosis in CTLs [43]. Therefore, the PD-1/PD-L1 immune checkpoint axis is a critical tool utilized by tumorigenic cells to avoid immune destruction, and thus it is imperative to treat cancer patients with therapies capable of blocking this axis [44]. 

## 3. Monoclonal Antibodies

In 1986, the first mAb, known as muromonab-CD3, was Food and Drug Administration (FDA) approved for the prevention of kidney transplant rejection, and thereafter more than a hundred additional mAbs have been approved for a broad range of ailments, including psoriasis, rheumatoid arthritis, macular degeneration, and cancer [15,46,47]. These antibodies fall under the Ig superfamily and are large glycoproteins capable of recognizing and binding to foreign or tumorigenic-specific antigens [48]. Antibodies are divided into five classes, including IgA, IgD, IgE, IgG and IgM, all of which are further differentiated based on the molecular weight (MW), charge, and the size and composition of the heavy chain [8].

IgG is the most common class of antibodies used in therapy and possesses two identical heavy chains (HC) and light chains (LC), each of which is composed of a constant and variable region [49,50]. In addition, the IgG class can be further subdivided into four subclasses (IgG1–IgG4), which are all 90% identical in terms of their amino acid profiles but differ with respect to their number of disulphide bonds, length of the hinge regions and the fragment crystallizable (Fc)-effector functions [51]. IgG antibodies exhibit three main Fc-effector functions, including antibody-dependent cellular phagocytosis (ADCP), complement-dependent cytotoxicity (CDC) and antibody-dependent cell-mediated cytotoxicity (ADCC) [52,53]. Approximately half of the mAbs used for cancer therapy exert anti-tumor effects via these Fc-mediated effector functions [51]. The remaining mAbs’ anti-tumor effects are exerted via different mechanisms, including the inhibition of angiogenesis, inhibition of tumor growth signals or activating the immune system by obstructing immune checkpoints [51].

The use of mAbs for targeted cancer therapy has increased significantly over the past decade and has led to remarkable clinical outcomes for a wide range of cancers [15]. Due to this, researchers have aimed to engineer a novel class of proteins known as Affimer proteins that exhibit comparable binding and specificity as mAbs in order to circumvent some of the manufacturing challenges associated with mAbs [54]. Affimer proteins provide distinct benefits, including enhanced stability across various conditions (e.g., temperature, pH), ease of production and scalability [55]. Despite the potential of Affimer proteins as an alternative, substantial research and optimization is required to attain comparable levels of efficacy, reliability, and widespread acceptance of mAbs [55]. Nonetheless, the most frequently used mAbs for cancer therapy are pembrolizumab, nivolumab, bevacizumab, trastuzumab and rituximab, which target proteins involved in tumorigenesis including PD-1, vascular endothelial growth factor A (VEGF-A), human epidermal growth factor receptor 2 (HER2) and CD20, respectively (Table 1) [15]. Furthermore, pembrolizumab and nivolumab are anti-PD-1 mAbs used for immune checkpoint blockade therapy and inhibit the PD-1/PD-L1 axis between T lymphocytes and tumorigenic cells, leading to the activation of CTLs and the induction of apoptosis in tumorigenic cells through T-cell-mediated cytotoxicity [13,36]. In addition, pembrolizumab and nivolumab are the most frequently used mAbs for cancer treatment and accounted for a combined revenue of USD 24.8 billion in 2021 alone, making pembrolizumab and nivolumab two of the most lucrative currently available drugs on the market [56]. Moreover, the high profits produced by these two mAbs are attributed to their ability to yield exceptional anti-tumor responses with limited side effects in cancer patients [57]. 

### 3.1. Immune Checkpoint Inhibitors

Immune checkpoint proteins play a major role in the progression of cancer and its ability to evade immune surveillance and thus anti-tumor immunity [13]. PD-1, PD-L1 and cytotoxic T lymphocyte antigen 4 (CTLA-4) are three proteins that impede the induction of T-cell-mediated cytotoxicity and subsequent immune responses in autoimmunity and pregnancy [20]. PD-1, PD-L1 and CTLA-4 are the most well-studied checkpoints involved in cancer progression today and are frequent biochemical targets for immune checkpoint blockade therapy [58]. Furthermore, CTLA-4 is a homolog of a co-stimulatory protein known as CD28, which functions to activate CTLs and promote survival by binding to CD80/CD86 on tumorigenic cells resulting in the generation of a co-inhibitory signal capable of preventing CTL activation via the stimulation of SHP2, which subsequently inhibits the PI3K/Akt signaling pathway [59,60].

Ipilimumab (Yervoy^®^ (Bristol-Myers Squibb, New York, NY, USA)) is an anti-CTLA-4 mAb which was the first ICI to be FDA approved, in 2011, for the treatment of metastatic melanoma [61]. This mAb laid the foundation of ICIs for cancer treatment and paved the way for the development of six additional ICIs, approved for more than nineteen types of cancer, including melanoma, NSCLC, and Hodgkin’s lymphoma [58]. Three of these ICIs target PD-L1, namely avelumab (Bavencio^®^ (EMD Serono Inc., Rockland, ME, USA)), atezolizumab (Tecentriq^®^ (Genentech, San Francisco, CA, USA)) and durvalumab (Imfinzi^®^ (Regeneron Pharmaceuticals, Tarrytown, NY, USA)), where the remaining three mAbs targeting PD-1 are pembrolizumab (Keytruda^®^ (Merck, NJ, USA)), nivolumab (OPDIVO^®^ (Bristol-Myers Squibb, New York, NY, USA)) and cemiplimab (Libtayo^®^ (Regeneron Pharmaceuticals, Tarrytown, NY, USA)) [58]. Overall, pembrolizumab and nivolumab generate superior clinical outcomes in a wider variety of cancer types when compared to the other ICIs, and are thus essential therapeutics within the current cancer therapy arsenal [48].

#### Pembrolizumab and Nivolumab

Pembrolizumab is a 149 kDa IgG 4 mAb marketed under the brand name Keytruda^®^ and is manufactured and sold by Merck (Branchburg, NJ, USA) [15,62]. Pembrolizumab was FDA approved on the 4th of September 2014 following promising results obtained from a clinical trial (KEYNOTE-001) evaluating the efficacy of an infusion consisting of 2 mg/kg or 10 mg/kg every 3 weeks for patients with metastatic or unresectable melanoma—for instance, pembrolizumab showed an overall response rate (ORR) of 33% [62,63]. Subsequently, pembrolizumab was approved for a further seventeen types of cancer known to frequently express high levels of PD-L1, including advanced Merkel cell carcinoma, TNBC, melanoma and Hodgkin’s lymphoma [62]. For first-line therapy, treatment with pembrolizumab proved to significantly increase survival rates when compared to other treatment options, as demonstrated by the administration of 200 mg pembrolizumab every 3 weeks, where observations included an increased survival rate to 31% in patients with metastatic head and neck squamous cell cancer (HNSCC) compared to the standard treatment (cetuximab, fluorouracil and platinum-based compounds) which only produced a 19% survival rate [62]. In addition, a randomized clinical trial comparing pembrolizumab and ipilimumab for the treatment of advanced melanoma found that patients receiving pembrolizumab had a 67% survival rate compared to a 60% survival rate in patients receiving ipilimumab [62]. Moreover, this study reported that pembrolizumab reduced the risk of disease progression by approximately 42% compared to ipilimumab [62].

The recommended dose of pembrolizumab is 200 mg every 3 weeks or 400 mg every 6 weeks and is administered as an intravenous infusion for 30 min; however, the dosage differs depending on cancer type and severity—for instance, in melanoma, a dosage of 2 mg/kg every 3 weeks is required [62,63,64,65,66]. The duration of treatment depends entirely on the patient’s response, although the usual duration is 24 months [66]. As of 2022, the current price for a 200 mg or 400 mg infusion is USD 10,683 or 21,367, respectively [62]. Therefore, cancer patients can expect to pay an exorbitant cost of over USD 370,000 for a full duration of treatment, and thus the majority of patients in LMICs cannot afford to access this treatment [3].

Nivolumab is another costly ICI, which is a 146 kDa IgG4 mAb marketed under the brand name OPDIVO^®^ and is produced and sold by Bristol-Myers Squibb (New York, NY, USA) [15] (Table 2). Nivolumab was FDA approved on the 22nd of December 2014 after proving effective for the treatment of patients with unresectable or metastatic melanoma who no longer responded to ipilimumab and B-Raf proto-oncogene (BRAF) inhibitors [58]. Subsequently, nivolumab was approved for an additional 11 types of cancer, including colorectal cancer and malignant pleural mesothelioma [67]. Several clinical trials have demonstrated that treatment with nivolumab significantly increases survival rates when used as a first-line therapy both as a single agent and in combination with ipilimumab [58]. Furthermore, treatment with exclusively nivolumab reduces cancer progression and therefore increases the chances of survival in patients, as demonstrated by a study that included 361 HNSCC patients that compared the effects of nivolumab (240 mg every 2 weeks) with standard therapy options, including docetaxel, cetuximab, and methotrexate [67]. The results from this study showed that 36% of patients receiving nivolumab and 17% of patients receiving the standard therapy options survived after one year of treatment [67]. Moreover, the combination of nivolumab and ipilimumab is frequently used to treat more than ten types of cancer, including renal cell carcinoma, hepatocellular carcinoma and colorectal cancer [68]. This combination therapy exhibited tremendous clinical success rates, shown in a clinical trial that included 605 patients with malignant pleural mesothelioma, where the combinational treatment was compared to platinum-based chemotherapy [67]. The results demonstrated that the combination of nivolumab and ipilimumab given at a dosage of 10 mg/mL and 5 mg/mL, respectively, reduced the risk of mortality by 26% in comparison to platinum-based chemotherapy and additionally resulted in a 23% survival rate versus a 15% survival rate obtained from platinum-based chemotherapy [67].

The recommended dosage of nivolumab is similar to pembrolizumab of either 240 mg every 2 weeks or 480 mg every 4 weeks and is intravenously administered over 30 min [67,68]. The typical treatment duration is between 12–24 months or until significant disease regression is observed or unacceptable toxicity has been noted [67]. The current price for a 240 mg or 480 mg infusion is USD 7194 and 14,389, respectively [67]. Thus, cancer patients should anticipate the minimum costs to be approximately USD 370,000, almost indistinguishable from the cost for a full duration of Keytruda® (Merck, NJ, USA). Unfortunately, the price of OPDIVO limits its availability in LMICs and leads to large financial burdens and distress in poverty-stricken cancer patients [3,17].

Although pembrolizumab and nivolumab have demonstrated remarkable clinical responses in a myriad of cancer types, a significant proportion of patients do not respond to these treatments, particularly those with “cold tumors”, characterized as tumors presenting with low T-cell infiltration [69]. Immunotherapies, such as ICIs, are often ineffective against cold tumors due to the limited infiltration of CTLs into the tumor, resulting in significantly lower response rates [69]. Hence, extensive research has focused on deciphering the molecular mechanisms underlying the development of cold tumors and identifying therapeutics that are capable of transforming cold tumors into responsive, hot tumors [70]. Various clinical trials, including NCT03301636 and NCT03066778, have explored new therapeutic modalities in combination with pembrolizumab or nivolumab to mitigate the limitations associated with these ICIs [71,72]. Furthermore, NCT03301636, a phase 2/3 clinical trial, investigated the concurrent administration of indoximod or placebo in combination with either pembrolizumab or nivolumab in adult patients diagnosed with unresectable stage III or stage IV malignant melanoma. Indoximod, an inhibitor of indoleamine 2,3-dioxygenase 1 (IDO1), is an immunometabolic adjuvant capable of enhancing immune cell infiltration within the tumor, and thereby effectively transforms cold tumors into a hot, immunologically active state [73]. Although the results of this study are still to be reported, the approach utilized in the study provides valuable insights into a novel combinational strategy that holds promise for enhancing the effectiveness of ICIs against cold tumors. Moreover, other modalities are being investigated, including epigenetic modification inhibitors, oncolytic viruses and photodynamic therapy, aiming to uncover additional avenues to enhance the efficacy of pembrolizumab and nivolumab in targeting cold tumors [69]. 

Despite the ongoing exploration for alternative therapeutic interventions to improve the efficacy of ICIs, the high cost of pembrolizumab and nivolumab remains a significant barrier, preventing the majority of the world’s population from accessing these treatments [16,17]. Moreover, the prohibitively high cost of these two highly beneficial therapies will persist, leaving millions of cancer patients unable to benefit from their anti-cancer effects, ultimately leading to increased mortality rates [74]. In spite of the significant efforts made by governmental agencies to promote the financial accessibility of these therapies, health insurance schemes in developing countries, unfortunately, fail to reach the intended underprivileged populations [75]. For this reason, it is a necessity for the price of these therapies to be significantly reduced; however, due to the costly mammalian production platform used, it is unlikely for these therapies’ prices to drop [76]. It is thus imperative to identify new cost-effective platforms for the production of pembrolizumab and nivolumab to mitigate the financial burden bought upon by these ICIs [16]. 

## 4. Traditional Manufacturing Methods

At present, approximately 95% of all mAbs are produced in mammalian cells since this manufacturing platform is capable of producing complex IgG mAbs indistinguishable from their human body counterparts [19]. However, alternative production methods, such as phage display, have been employed, resulting in the development of several FDA-approved mAbs, including adalimumab and ramucirumab [15]. Antibody phage display is an effective in vitro selection technique that enables the identification of high-affinity antibodies targeting a diverse range of antigens [77]. The antibody phage display workflow involves the isolation of mRNA encoding the variable heavy (VH) and variable light (VL) chains of the antibody from peripheral blood mononuclear cells (PBMCs), which is subsequently reverse transcribed into complementary DNA (cDNA) [77]. The cDNA is then amplified through a polymerase chain reaction (PCR) using a specific set of primers to create a diverse repertoire of Igs [15,77]. Thereafter, the PCR fragments are cloned into a phagemid, which is constructed to express the VH and VL chains as single-chain variable fragments (scFv) fused to the pIII capsid protein on the filamentous bacteriophage. Following this, the phagemid is electroporated into competent Escherichia coli (*E. coli*) cells together with the helper phage genes needed to produce complete bacteriophages [77]. Finally, the antibody phage library undergoes multiple cycles of screening known as biopanning to identify high-affinity antibodies [15,77]. Overall, phage display has been demonstrated to be an effective methodology for mAb production; however, the cost associated with constructing a phage display library is higher compared to traditional mammalian expression platforms [78]. In addition, mammalian platforms are favorable for mAb production since this system ensures that the correct post-translational modifications, including protein folding and N-linked glycosylation, are fulfilled [8]. Furthermore, the N-linked glycosylation of mAbs includes the addition of multiple sugar moieties consisting of N-acetylglucosamine (GlcNAc), mannose, fucose and galactose through the covalent attachment to an amide nitrogen on an asparagine residue in the endoplasmic reticulum (ER) [79,80]. Altogether, the glycosylation of mAbs is critical to ensure that the correct structure, stability and biological functions are acquired [79]. Thus, the use of mammalian cells for the production of mAbs is considered to be a gold standard and has undergone extensive regulatory approvals to ensure good manufacturing practice (GMP) [16,19].

Hybridoma technology is one of the most frequently used mammalian production platforms for mAbs and was first developed in 1975 by Georges Köhler and Cesar Milstein, who went on to win the 1984 Nobel Prize in Physiology or Medicine [81,82]. Moreover, the traditional hybridoma technique involves the initial immunization of mice with a specific target antigen in order to stimulate an immune response and therefore generate antibodies against the antigen [15,82]. Subsequently, B-lymphocytes are isolated from the spleen of the mice and are fused with an immortal myeloma cell line to generate hybridoma cells that continuously produce mAbs [82,83]. The hybridoma technology was at the forefront of mAb production for multiple years; however, due its inability to produce stable antibodies in large quantities, there was a major shift to the utilization of recombinant DNA technology for the large-scale manufacturing of mAbs [83]. Pembrolizumab and nivolumab are both produced in Chinese hamster ovary (CHO) cells, which are currently the favored mammalian cell expression system for recombinant mAb production employed by Merck and Bristol-Myers Squibb [62,67,84].

Pembrolizumab and nivolumab are humanized (-zumab) and human (-umab) mAbs, respectively, both of which are produced by the initial immunization of mice with the human target protein (PD-1) to trigger an immune response [15,85]. Subsequently, antibodies specific to PD-1 are identified, and the DNA encoding the LC and HC are extracted from B-lymphocytes in the spleen and are stably transfected into CHO cells, generating a recombinant mAb-producing cell line [85]. The production of human mAbs (nivolumab) differs from that of humanized mAbs in that human mAbs are produced in transgenic mice, which have been genetically engineered through the replacement of murine Ig genes with human ones [15,85]. Ultimately, these transgenic mice are capable of synthesizing fully human mAbs, and the gene sequence of these mAbs is directly transfected into CHO cells, whereas humanized mAbs are produced in wild-type mice, leading to the production of purely murine mAbs [15,85,86,87]. However, instead of directly transfecting the genes of the murine mAbs into CHO cells, the DNA must first undergo complementarity determining region (CDR) grafting, whereby the CDRs of the mAb variable region are inserted into a human framework sequence using recombinant DNA technology [15,87,88]. Subsequently, the framework is stably transfected into CHO cells, leading to the generation of humanized mAbs with an optimized binding activity to the human target protein compared to murine mAbs [87]. Nonetheless, the CHO cell production platforms used for the biomanufacturing of pembrolizumab and nivolumab possess several disadvantages, including high manufacturing and maintenance costs, safety concerns and laborious upstream processes [8,16,89]. Therefore, the high price associated with these two therapies is greatly attributed to the manufacturing platform employed and reflects a need for an alternative production method that is superior to mammalian cell systems with regard to cost, safety and scalability [76]. However, it is worth noting that the high price for these therapies is not solely due to the production platform utilized, but is also due to the costly regulatory path and intellectual property rights.

## 5. Molecular Farming

Molecular farming is the production of recombinant proteins in plants and has gained immense interest in the biotechnology sector since it offers a novel platform that is straightforward, rapid and scalable [90]. In addition, plants have the ability to be propagated indefinitely, providing low-cost biomass production that can be used for the large-scale manufacturing of mAbs [8]. The first mAb produced in Nicotiana benthamiana that gained worldwide attention was ZMapp, a triple mAb cocktail, which was the first drug experimentally tested against the Ebola virus in 2014 [91]. Overall, ZMapp revealed that the molecular farming of mAbs is a viable platform capable of rapidly producing mAbs at high yields [92]. Thus, the utilization of molecular farming for the mass production of anti-cancer mAbs has become an attractive field of research [8]. However, due to concerns regarding the ability of plant-produced mAbs to exhibit the same structure, N-linked glycosylation and binding activity, the commercialization of these mAbs has remained futile [8]. Nonetheless, multiple research institutions have removed these concerns by showing that molecular farming can be used for the large-scale manufacturing of mAbs under GMP regulations [93]. In addition, these institutions have shown that plant-produced mAbs meet the quality standards specified by the FDA in terms of structure, potency and purity [93].

Recently, studies conducted in 2019 and 2021 demonstrated that the transient expression of pembrolizumab and nivolumab in Nicotiana benthamiana is a viable manufacturing platform with the possibility to significantly reduce the price of these therapeutics [16,17]. Nicotiana benthamiana is the most extensively used plant species for the stable and transient expression of mAbs due to its fast growth rate and ease of genetic manipulation [92]. Furthermore, transient expression differs from stable expression in that the transgenes are not integrated into the plant cell genome, resulting in the loss of the expression vector after several replication cycles [94]. Nonetheless, transient expression is an efficient method for the production of mAbs and has been demonstrated to produce over 350 mg/kg leaf material in no more than 4 days [16,95]. Transiently expressing mAbs in Nicotiana benthamiana is accomplished by leveraging the ability of Agrobacterium tumefaciens to transfer a particular segment of DNA (T-DNA) from the tumor-inducing (Ti) plasmid into plant cells [96,97]. Hence, the target genes encoding the light and heavy chains of pembrolizumab and nivolumab can be inserted into the T-DNA region of an expression vector, which can then be transformed into plant cells following agroinfiltration [98]. Subsequently, the plants are grown and cultivated in GMP indoor hydroponic growth facilities with optimal climatic and light conditions to ensure high levels of protein expression [92,99]. Finally, the plants undergo multiple extraction and purification processes, including blending, centrifugation and affinity chromatography, to retrieve the mAbs [16,17].

### 5.1. Vector Construction

The design and subsequent construction of expression vectors for agrobacterium-mediated transformation is imperative to ensure the optimum transient expression of the genes encoding the HC and LC of pembrolizumab or nivolumab [16,17]. The gene fragments of the HC and LC first undergo codon optimization using the in silico GeneArt^®^ software supplied by Invitrogen (ThermoFisher Scientific, Waltham, MA, USA) and are subsequently synthesized and amplified by means of polymerase chain reaction (PCR) [16,17,89]. Thereafter, the HC and LC genes of pembrolizumab and nivolumab are digested with XbaI and SacI, and ligated into a pBYR2e geminiviral vector harboring a T-DNA region [16,17,89]. The vectors are then transformed into Agrobacterium tumefaciens strain GV3101 via electroporation, which involves the use of high-voltage electric shocks to create pores within the bacterial cell membrane through which the expression vectors can pass [16,89,100]. Subsequently, the cells are grown overnight, followed by centrifugation and finally resuspension in the agroinfiltration buffer containing 10 mM 2-(N-morpholino) ethanesulfonic acid (MES) and 10 mM MgSO4 at a pH 5.5 [16,17,89].

### 5.2. Agroinfiltration and Plant Growth

To successfully agroinfiltrate expression vectors into Nicotiana benthamiana leaf cells, it is crucial to first complete an optical density (OD) measurement to ensure that the correct number of Agrobacterium tumefaciens cells are present within the agroinfiltration buffer [101,102]. The most frequent way to perform an OD measurement is by determining the absorbance of the cell suspension at a wavelength of 600 nm using a spectrophotometer, which allows for the rapid and precise quantification of cell number [102,103]. Furthermore, a final OD_600_ of 0.2, which equates to 1.6 × 10^8^ cells/mL, is required for the successful agroinfiltration and delivery of genes encoding the HC and LC of pembrolizumab or nivolumab into plant cells [16,17,89,104]. The agrobacterium cell suspension is then infiltrated into the leaves of 6–8-week-old wild-type Nicotiana benthamiana plants [16,89,105]. Finally, the plants are either grown in greenhouses or indoor growing facilities, which are under strictly controlled environmental conditions to ensure that the correct temperature, humidity, light exposure and sterility are maintained for optimal protein yields [16,106]. Additionally, the ideal environmental conditions for the mass production of pembrolizumab and nivolumab in plants is at a temperature of 28 °C, a 70% humidity, and a 16 h light/8 h dark cycle at a light intensity between 80–100 μmol m^−2^ s^−1^ [16,106,107,108,109]. The plants are grown under these conditions for a period of 4 days following agroinfiltration and thereafter undergo the extraction and purification process to obtain purified pembrolizumab and nivolumab [16,17,89].

### 5.3. Purification

The purification of pembrolizumab and nivolumab from plants is initiated by first removing the agroinfiltrated leaves from the plant, followed by the homogenization of the leaves with 1X phosphate buffer solution (PBS) in an electronic blender [8,16,17]. Blending disrupts the plant cell wall by shear force, leading to the release of the intracellular contents, resulting in the formation of homogenate [8]. Subsequently, the homogenate is centrifuged at approximately 26,000× *g* for 40 min at 4 °C to remove cell debris and is further filtered through a membrane filter with a pore size of 0.45 µm [16,17]. The supernatant is then purified using protein A affinity chromatography, which is a highly efficient purification technique used to capture and purify IgG mAbs due to the high affinity of the protein A bead column for the Fc region [16,17,110,111]. Finally, the recombinant anti-PD-1 mAbs are removed from the column by washing with 1X PBS, and further eluted and neutralized with 0.1 M glycine and 1.5 mM Tris(hydroxymethyl)aminomethane hydrochloride (Tris-HCl), respectively. Finally, the concentrations of pembrolizumab and nivolumab are determined using enzyme-linked immunosorbent assay (ELISA) and are quantified as µg/g of fresh leaf weight (FLW) [16,17,89]. Previous studies have shown that the transient expression of pembrolizumab and nivolumab in wild-type Nicotiana benthamiana leaves produce more than 340 µg/g and 140 µg/g FLW, respectively [16,17]. Overall, this is equivalent to a total of 340 mg/kg and 140 mg/kg FLW, which equates to approximately USD 18,000 and 4200 of pembrolizumab and nivolumab in 1 kg of leaves, respectively. Ultimately, this demonstrates that the utilization of molecular farming for the production of pembrolizumab and nivolumab is a viable platform that can be potentially implemented in LMICs to increase the accessibility of these ICIs. However, before the commercialization of plant-produced pembrolizumab and nivolumab can take place, it is crucial to complete both in vitro and in vivo testing to ensure that the same structure and activity are exhibited in those that are already commercially available [16,17].

### 5.4. Structural and Functional Assays

Following the purification and quantification of plant-produced pembrolizumab and nivolumab, multiple structural (physicochemical) and functional in vitro assays need to be conducted [16,17]. The physicochemical assays required to ensure that the structural characteristics of the purified anti-PD-1 mAbs are similar to the commercially available pembrolizumab and nivolumab include sodium dodecyl sulphate-polyacrylamide gel electrophoresis (SDS-PAGE), western blot, circular dichroism (CD) spectroscopy, nuclear magnetic resonance (NMR) spectroscopy and liquid chromatography-electrospray ionization-mass spectrometry (LC-ESI-MS) [16,17]. Furthermore, SDS-PAGE and western blot are conducted to determine whether the plant-produced mAbs are correctly assembled and have a similar MW compared to the commercial pembrolizumab and nivolumab produced in CHO cells [16,17,89]. In addition, the secondary and tertiary structures of the anti-PD-1 mAbs are determined using CD and NMR spectroscopy, respectively [16,17,112,113]. Finally, LC-ESI-MS is used to determine the N-glycosylation profile of the plant-produced pembrolizumab and nivolumab and to confirm whether this profile is similar to that commercially produced in CHO cells [16,17,114]. Altogether, Phakham et al. revealed that transiently expressing pembrolizumab and nivolumab in Nicotiana benthamiana leaves leads to anti-PD-1 mAbs that assemble into the correct tetrameric form, have similar secondary and tertiary structures, have slightly different N-glycosylation profiles, and have remarkably similar MWs. For instance, plant-produced pembrolizumab had a MW of 150 kDa, which is only 1 kDa different compared to commercial pembrolizumab (149 kDa) [16,17]. Moreover, the assays required to determine the functional characteristics of the anti-PD-1 mAbs include ELISA, surface plasmon resonance (SPR) and luciferase reporter assay, which are used to investigate the PD-1 binding affinity, kinetics and PD-1/PD-L1 inhibitory activity, respectively. Phakham et al. reported that there were no significant differences between the binding affinity and kinetics of the plant-derived mAbs to PD-1 when compared to the commercial mammalian-produced pembrolizumab and nivolumab [16,17]. Finally, the plant-produced pembrolizumab and nivolumab demonstrated the crucial ability to inhibit the binding of PD-1 to PD-L1 in a dose-dependent manner with a half-maximal effective concentration (EC50) of 147.2 ng/mL and 496 ng/mL, respectively, when compared to commercial pembrolizumab and nivolumab, which had EC_50s_ of 146.7 ng/mL and 544 ng/mL, respectively [16,17]. On the whole, Phakham et al. were the first to successfully demonstrate that the transient expression of pembrolizumab and nivolumab in Nicotiana benthamiana leaves is a rapid, simple and cost-effective production platform capable of producing mAbs that exhibit the correct assembly, molecular weight, structure, binding affinity, kinetics and PD-1/PD-L1 inhibitory activity [16,17]. However, significant research is still required to optimize the N-linked glycosylation of these plant-derived mAbs.

## 6. Advantages and Future Prospects

The molecular farming of pembrolizumab and nivolumab offers unique advantages over mammalian production platforms, as it is a rapid and economical method that can be easily upscaled to produce GMP-compliant facilities for large- and small-scale manufacturing [115,116]. Furthermore, it has been estimated that molecular farming can be used to produce recombinant proteins at approximately 0.1% of the cost of mammalian cell platforms, provided that significant yields are maintained [117]. It is worth noting that transgenic plants are prohibited in several countries due to bioethical concerns; however, by utilizing the transient expression method, no transgenic plants are produced, and thus the regulatory issues and public concerns associated with genetically modified organisms (GMO) are alleviated [17,118]. Moreover, in 2015, the first regulatory approval for the use of plant-derived mAbs was completed following the results obtained from a randomized, double-blind, placebo, phase 1 clinical trial investigating the vaginal administration of the plant-produced P2G12 mAb for the prevention of human immunodeficiency virus-1 (HIV-1) infection [119]. Notably, no anti-P2G12 antibodies were detected in serum or vaginal fluid, regardless of the dosage administered, unequivocally demonstrating that plant-produced mAbs exhibit near to no immunogenicity in humans [119]. However, the literature is limited referring to the immunogenic properties of plant-produced mAbs; thus, further research is required to validate these results [119]. Overall, this study confirmed that plant-produced P2G12 mAbs were safe and well-tolerated in patients and were able to meet the same quality criteria concerning structure, half-life, stability, and HIV-1 neutralization activity when compared to their CHO-cell-produced counterparts (C2G12) [119]. Ultimately, this study suggests that similar results would be obtained with plant-produced pembrolizumab and nivolumab in cancer patients; however, as only in vitro studies have been completed, there is a necessity for future research focusing on analyzing the in vivo effects.

Despite the significant advancements made within the field of molecular farming, the translation of plant-derived mAbs to market is a timely issue due to the limited amount of funding and resources allocated to molecular farming research, which results in the restriction of the commercial utilization of plant-based platforms for mAb production in LMICs [120]. Currently, there are no plant-produced anti-cancer mAbs approved for clinical use; however, with the biotechnology market predicted to increase at a compound annual growth rate (CAGR) of 15.83% by 2028, it is expected that research focusing on plant-produced antibodies, including pembrolizumab and nivolumab, will significantly increase, potentially leading to their clinical use for cancer immunotherapy [121].

## 7. Conclusions

Pembrolizumab and nivolumab are the most frequently used ICIs on the market today and are used to treat a plethora of cancers, including melanoma, Hodgkin lymphoma, colorectal, breast and lung cancer [15,16,17]. These two ICIs inhibit the PD-1/PD-L1 immune checkpoint leading to the activation of CTLs and the induction of apoptosis in tumorigenic cells through T-cell-mediated cytotoxicity [11,13]. Literature indicates that pembrolizumab and nivolumab significantly increase the survival rates of patients with a wide range of cancer types; however, due to the price of these two therapies being well above USD 300,000 per treatment regime, their accessibility to patients in LMICs is severely limited [3]. The mammalian production platform utilized for the manufacturing of pembrolizumab and nivolumab is a contributing factor to their extortionate price, and with the incidence of cancer expected to increase by 47% in 2040, it is imperative to employ alternative production platforms [1,16,17]. Molecular farming is one such platform with the potential to significantly reduce the capital needed for these two therapies [16,17]. The transient expression of pembrolizumab and nivolumab in Nicotiana benthamiana leaves is a straightforward, rapid, scalable and cost-effective platform capable of producing a total of 340 mg/kg and 140 mg/kg FLW, respectively [16,17]. Furthermore, this was calculated to equate to approximately USD 18,000 and 4200 worth of pembrolizumab and nivolumab in 1 kg of leaves, respectively.

In conclusion, this review demonstrates that the molecular farming of pembrolizumab and nivolumab is a viable manufacturing platform potentially capable of bridging the accessibility gap in LMICs; however, much research is still required to optimize this platform and to determine the in vivo effects. In addition, this review aids in the further understanding of the PD-1/PD-L1 axis, the mechanism of action of PD-1 ICIs, the mammalian mAb production platform, and finally, the methods utilized for plant-based mAb manufacturing.

## Figures and Tables

**Table 1:** Most frequently used mAbs for the treatment of cancer [15].

mAb	Target	FDA Approval Year	Main Indications	Mechanism of Action
Pembrolizumab (Keytruda^®^, Merck, NJ, USA)	PD-1	2014	Melanoma, head and neck cancer, NSCLC, lymphoma, kidney, breast, esophageal, colorectal, endometrial, urothelial and cervical cancer.	Inhibition of PD-1/PD-L1 immune checkpoint
Nivolumab (OPDVIO^®,^ Bristol-Myers Squibb, NY, USA)	PD-1	2014	Melanoma, head and neck cancer, NSCLC, pleural mesothelioma, lymphoma, kidney, liver, colorectal, stomach, esophageal and urothelial cancer.	Inhibition of PD-1/PD-L1 immune checkpoint
Bevacizumab (Avastin^®^, San Francisco, CA, USA)	VEGF-A	2004	Colorectal cancer, NSCLC, renal cell carcinoma, glioblastoma, breast, ovarian and cervical cancer	Inhibition of angiogenesis
Trastuzumab (Herceptin^®^, San Francisco, CA, USA)	HER2	1998	Breast cancer, esophageal cancer and gastric cancer	Inhibition of HER2 mediated cell signaling pathways
Rituximab (Rituxan^®^, San Francisco, CA, USA)	CD20	1997	Non-Hodgkin’s lymphoma and chronic lymphocytic leukemia	Activation of Fc-effector functions (ADCC, ADCP and CDC)

**Table 2:** Structural and functional comparison of pembrolizumab and nivolumab [15,62,67].

mAb	Structure	Expression	Price (2022–2023)	Average Duration of Course
Pembrolizumab	Humanized IgG4	Recombinant Chinese hamster ovary (CHO) cells	USD 10,683 per 200 mg infusion every 3 weeks	2 years
Nivolumab	Human IgG4	Recombinant CHO cells	USD 7194 per 240 mg infusion every 2 weeks	2 years

## Data Availability

Data is contained within the article.
